# Prevention of lethal murine pancreas ischemia reperfusion injury is specific for tetrahydrobiopterin

**DOI:** 10.1111/j.1432-2277.2012.01530.x

**Published:** 2012-07-17

**Authors:** Manuel Maglione, Benno Cardini, Rupert Oberhuber, Katrin Watschinger, Marcel Jenny, Johanna Gostner, Martin Hermann, Peter Obrist, Raimund Margreiter, Johann Pratschke, Gerald Brandacher, Ernst R Werner

**Affiliations:** 1 Center of Operative Medicine, Department of Visceral, Transplant and Thoracic Surgery, Innsbruck Medical UniversityInnsbruck, Austria; 2 Division of Biological Chemistry, Biocenter, Innsbruck Medical UniversityInnsbruck, Austria; 3 Division of Medical Biochemistry, Biocenter, Innsbruck Medical UniversityInnsbruck, Austria; 4 Department of Anesthesiology and Critical Care Medicine, Innsbruck Medical UniversityInnsbruck, Austria; 5 Innsbruck Medical University, Department of Pediatrics II, Innsbruck Medical UniversityInnsbruck, Austria; 6 Institute of Pathology, St. Vinzenz KHZams, Austria

**Keywords:** experimental transplantation, ischemia reperfusion injury, nitric oxide, nitric oxide synthase, organ preservation and procurement, pancreas, tetrahydrobiopterin

## Abstract

Tetrahydrobiopterin has been shown to efficiently abrogate ischemia reperfusion injury (IRI). However, it is unclear, whether its beneficial action relies on cofactor activity of one of the five known tetrahydrobiopterin-dependent reactions or on its antioxidative capacity. We therefore compared tetrahydrobiopterin with the pterin derivate tetrahydroneopterin (similar biochemical properties, but no nitric oxide synthase cofactor activity) and the antioxidants vitamin C and 5-methyltetrahydrofolate. Donor mice were pretreated with tetrahydrobiopterin, tetrahydroneopterin, vitamin C, or 5-methyltetrahydrofolate. Pancreatic grafts were subjected to 16-h cold ischemia time and implanted in syngeneic recipients. Untreated and nontransplanted animals served as controls. Following 2-h reperfusion, microcirculation was analyzed by intravital fluorescence microscopy. Graft damage was assessed by histology and nitrotyrosine immunostaining, and tetrahydrobiopterin levels were determined by HPLC. Recipient survival served as ultimate readout. Prolonged cold ischemia time resulted in microcirculatory breakdown. Only tetrahydrobiopterin pretreatment succeeded to preserve the capillary net, whereas all other compounds showed no beneficial effects. Along with increased intragraft tetrahydrobiopterin levels during recovery and implantation, only tetrahydrobiopterin pretreatment led to significant reduction of IRI-related parenchymal damage enabling recipient survival. These results show a striking superiority of tetrahydrobiopterin in preventing lethal IRI compared with related compounds and suggest nitric oxide synthases as treatment target.

## Introduction

Simultaneous kidney pancreas transplantation represents nowadays the therapy of choice for patients suffering from insulin-dependent diabetes mellitus and end-stage renal disease to achieve long-term normoglycemia with insulin independence, as well as a better quality of life [1,2]. Despite important progresses in surgical techniques and immunosuppression, ischemia-reperfusion injury (IRI) still represents a heavy burden in solid organ transplantation as its severity grade strongly influences outcomes [3,4]. IRI-associated graft pancreatitis remains a major quest following pancreas transplantation. According to the International Pancreas Transplant Registry (IPTR), graft pancreatitis is a major risk factor for graft thrombosis, which is one of the leading causes of early graft losses [1,5–7].

Recently, we were able to show in a mouse model that pretreatment of the donor with a single application of (6R)-5,6,7,8-tetrahydro-L-biopterin (BH4) abrogated IRI in pancreatic isografts. While occurrence of IRI in this model is lethal, donor pretreatment with BH4 resulted in indefinite recipient and graft survival [8]. BH4 is an essential cofactor for all three nitric oxide synthase (NOS) isoforms (endothelial, neuronal, and inducible), for aromatic amino acid hydroxylases, and for alkylglycerol monooxygenase [9–11], and also acts as a potent antioxidative agent, which is depleted during oxidative stress, like IRI condition [12]. Suboptimal concentrations of this cofactor lead to “uncoupling” of NOS with decreased production of NO and increased reduction of oxygen, generating superoxide anions and hydrogen peroxide, and resulting ultimately in tissue damage [13]. Despite various experimental studies observing prevention of IRI-associated graft dysfunction by BH4 supplementation, the mechanism is still unknown [14,15].

In this study, we investigated whether the beneficial effects observed are specific for BH4 by testing it head-to-head with other compounds, which were selected in a way that allows an allusion to the possible target of BH4 (Table 1) [16–20]. If the mode of action relied on antioxidative capacity, all compounds should protect the graft. However, if it required stimulation of an aromatic amino acid hydroxylase or alkylglycerol monooxygenase, only BH4 and tetrahydroneopterin (NH4) would be active. If NOS cofactor activity alone was required, no compound except BH4 should lead to prevention of ischemia-reperfusion injury. In particular, NH4 should be ineffective in this case as it shows no NOS cofactor activity in concentrations comparable to BH4 [21].

**1:** Biochemical properties of agents tested.

	BH4	NH4	VitC	5-MTHF
Antioxidative action	+	+	+	+
Phenylalanine hydroxylase cofactor activity	+	+	−	−
Tyrosine hydroxylase cofactor activity	+	+	−	−
Tryptophan hydroxylase cofactor activity	+	+	−	−
Alkylglycerol monooxygenase cofactor activity	+	+	−	−
Nitric oxide synthase cofactor activity	+	−	−	−

Summary of biochemical properties of the compounds used to treat ischemia-reperfusion injury in this study. While all compounds have high antioxidative potential, only BH4 can stimulate all known BH4-dependent enzymes. NH4, in contrast, cannot stimulate nitric oxide synthases.

## Material and Methods

### Animals

Ten- to twelve-week-old male C57BL6 (H2b) mice obtained from Harlan–Winkelmann Co. (Borchen, Germany) were used as syngeneic donor–recipient pairs. Animals were housed under standard conditions at the animal centre of Innsbruck Medical University and given mouse chow and water ad libitum. According to the ‘Principles of Laboratory Animal Care’ formulated by the National Society for Medical Research and the ‘Guide for the Care and Use of Laboratory Animals’ prepared by the National Academy of Sciences and published by the National Institutes of Health (NIH Publication No. 86-23, revised 1985), animals received humane care. Experiments were approved by the Austrian Federal Ministry for Education, Arts and Culture (BMWF-66.011/0133-II/10b/2008).

### Pancreas transplantation

Anesthesia was performed with an intramuscular injection of 100 mg/kg ketamine hydrochloride (Ketavet®, Pharmacia GmbH, Erlangen, Germany) and 10–15 mg/kg xylazine (Xylasol®, Dr. E. Gräub AG, Bern, Switzerland). Surgical procedures were carried out under clean conditions as previously described. In brief, through a midline incision, pancreas were recovered together with the spleen to avoid any contact with the graft. Exocrine secretion was managed by ligation of the choledocho-pancreatic duct; therefore, no exocrine drainage is necessary. In this model, almost normal islet and acinar architecture is found on postoperative day 10, without any signs of inflammation. Grafts were placed in the left cervical region of the recipient and revascularized through anastomosis with the recipient’s common carotid artery and the recipient’s external jugular vein. Before revascularization, the spleen was removed [22]. Postoperatively, 4 mg/kg carprofen were administered subcutaneously as an analgesic.

### Experimental design

All grafts were subjected to 16-h cold ischemia time. For graft recovery, perfusion solution Custodiol® (HTK, Dr. Franz Köhler Chemie GmbH, Alsbach-Hähnlein, Germany) was used. The experimental design consisted of six groups (*n* = 5 per group). Group 1, untreated; group 2, BH4 50 mg/kg; group 3, NH4 50 mg/kg; group 4, vitamin C (VitC) 350 mg/kg; group 5, 5-methyl tetrahydrofolate (5-MTHF) 50 mg/kg. Each treatment consisted of single-dose pretreatment of the donor 2 min before pancreas recovery. No treatment was given to recipient animals. Group 6 served as nontransplanted controls with mice subjected to laparotomy only. I.m. application was chosen because of its suitability in this model [15,23] and because of the rapid uptake of pteridines shown previously [24]. Two hours following reperfusion, grafts were exposed for confocal intravital fluorescence microscopy and finally retrieved for histopathological, immunohistochemical analyses, and intragraft BH4 tissue levels. Furthermore, intrapancreatic BH4 levels were measured at the time of retrieval, after cold ischemia time and before reperfusion (additional animals, *n* = 3 per time point and group).

In separately transplanted animals, recipients receiving grafts with different treatment compounds (BH4, NH4, VitC and 5-MTHF) as well as untreated grafts (*n* = 5 per group) were tested for survival [12]. In total, 155 mice were used for this study.

BH4 [(6R)-5,6,7,8-tetrahydro-L-biopterin dihydrochloride], NH4 [(6R,S)-5,6,7,8-tetrahydro-D-neopterin dihydrochloride], and 5-MTHF [(6S)-5-methyl-5,6,7,8-tetrahydrofolic acid, calcium salt] were obtained from Schircks Laboratories, Jona, Switzerland. VitC was obtained from Carl Roth GmbH + Co. KG, Karlsruhe, Germany.

### Confocal intravital fluorescence microscopy

Confocal intravital fluorescence microscopy was used for analysis of graft microcirculation. The mean functional capillary density (FCD), defined as the length of all blood cell perfused nutritive capillaries per observation area, and the mean capillary diameter (CD), defined as mean value of the three largest capillaries per observation area, were assessed.

Following graft exposure, 0.3 ml of a 0.4% fluorescein-isothiocyanate (FITC)-labeled dextran (MW 150.000; Sigma Aldrich, Vienna, Austria) were injected via the penile vein. Confocal microscopy and quantitative image analysis were performed as previously described [8].

### Histopathology

Grafts were fixed in 10% formaldehyde for 24 h, embedded in paraffin, and stained with hematoxylin and eosin (H&E). For quantification purposes, the semiquantitative pancreatitis Schmidt score was adopted, which divides parenchymal damage into four categories: interstitial edema, acinar necrosis, hemorrhage and fat necrosis, and inflammatory infiltrates. The score for each category ranges from 0 to 4 [25]. Tissue specimens were retrieved from the head of the transplanted pancreas.

### Nitrotyrosine Immunohistochemistry (IHC)

Graft sections were cut from paraffin blocks, mounted on slips, and the paraffin removed by heating in citrate buffer, pH 6.0. Endogenous peroxidase was blocked with 0.3% hydrogen peroxide. Nitrotyrosine-IHC was then performed in a diamonbenzidinetetrahydrochloride (DAB) autostainer (DAKO, Copenhagen, Denmark), using an anti-nitrotyrosine rat polyclonal antibody from Upstate Biotechnology (Lake Placid, NY, USA) at 1:100 dilution. For staining, secondary antibody peroxidase-labeled polymer and 3,3′-DAB were used. Haemalaun blue or methyl green was used for counterstaining. Specimens for immunohistochemistry analyses were retrieved from the pancreatic head.

### Biopterin and neopterin tissue levels

Intragraft biopterin concentrations were obtained by iodine oxidation of tissue supernatants in acid and base by a method modified from Fukushima and Nixon [8], HPLC separation of 20 μl of sample on a Nucleosil 10 SA column (250 mm long, 4 mm i.d., Macherey-Nagl, Düren, Germany), elution at 1.5 ml/min with 50 mM potassium phosphate buffer, pH 3.0, and fluorescence detection (excitation 350 nm, emission 440 nm). BH4 concentrations were calculated as difference of biopterin concentration under acidic and basic oxidation conditions.

Neopterin concentrations were quantified using the same protocol. We tested recovery of 7,8-dihydroneopterin and 5,6,7,8-tetrahydroneopterin after oxidation in acid and alkali and found that, unlike previous findings [21], dihydroneopterin was even more stable in alkaline oxidation (recovery: 80.0 ± 4.2%) than in acidic condition (recovery: 52.3 ± 6.5%). Recovery of tetrahydroneopterin in acidic and alkaline oxidation was 63.9 ± 4.3% and 4.2 ± 1.2%, respectively. Neopterin measurements were not corrected for the recovery and therefore the data present a measure for the minimum amount of tetrahydroneopterin present in the tissues.

To avoid bias because of possible intrapancreatic variation of the BH4 concentration, specimens for this analysis were always retrieved from the pancreatic tail.

### Survival analysis

The four different treatment compounds were tested for recipient survival and compared with untreated animals over an observation time of up to 50 days.

### Oxygen Radical Antioxidant Capacity (ORAC) assay

The capacity of the tested substances to scavenge peroxyl radicals was evaluated using the ORAC assay [26]. Briefly, the final assay mixture (0.2 ml total volume) contained 6.3 × 10^−8^M fluorescein (AnaSpec, Fremont, CA, USA) as a target of free radical attack and 1.9 × 10^−2^M 2,2′-azobis-(2-amidinopropane) dihydrochloride (AAPH, Wako Chemicals, Neuss, Germany), which decomposes in aqueous solutions at 37 °C and generates alkyl radicals (R′•) that are converted into the corresponding peroxyl radicals (R′OO•) in the presence of oxygen. Phosphate buffer (75mM) served as a blank, and 6-hydroxy-2,5,7,8-tetramethylchroman-2-carboxylic acid (Trolox, a synthetic water-soluble analog of vitamin E, Sigma-Aldrich, Vienna, Austria) was used as the control standard. The fluorescence was recorded at 37 °C by a fluorometer (Fluoroscan Ascent, Labsystems, Erlangen, Germany; 485 nm/535 nm). The decrease of fluorescence was measured in minute intervals after the addition of AAPH. Final results were calculated using the differences of areas under the fluorescein decay curves (AUC) between the blank and a sample, and were expressed as micromoles of Trolox equivalent per micromole of antioxidant (μmol TE/μmol).

### Measurement of influence on intracellular reactive oxygen species action

Influence of compounds on intracellular reactive oxygen species (ROS) levels was monitored by using 2′,7′-dichlorofluorescein diactetate (DCFH-DA, Sigma, Austria) [27]. In brief, 4.5 × 10^4^ cells/100 μl/well HepG2 cells were seeded in 96-well microplates. After 24 h, the cells were washed twice with Hank’s buffered salt solution (HBSS), which was followed by the treatment with 100 μl of 25 μM DCFH-DA dissolved in HBSS for 1 h. Subsequently, cells were either left untreated or treated with solvent (0.5% DMSO v/v), quercetin (10 μM) as a positive control or increasing doses of ascorbate, BH4, 5-MTHF, or NH4, for 1 h. Thereafter, the cells were washed twice with HBSS and treated with 600 μM AAPH (Wako Chemicals, Germany) dissolved in HBSS for 45 min at 37 °C. The fluorescence of DCF (excitation 485 nm, emission 538 nm) was determined using a Fluoroskan Ascent FL plate reader (Thermo Labsystems, Franklin, MA, USA).

### Statistics

Results are expressed as mean ± standard deviation. Statistical analysis was performed with GraphPad Prism 5 (GraphPad Software, La Jolla, CA, USA). When comparing multiple groups Kruskal–Wallis test was used. If statistical significance was achieved, all pairs were compared among each other using the Mann–Whitney-*U* test and the Bonferroni correction. Kaplan–Meier curve was used for survival analyses and groups were compared using the log rank test. A *P* value of <0.05 was considered to be of statistical significance (NS = not significant).

## Results

### Graft microcirculation

Sixteen-hours cold ischemia time resulted in severe microcirculatory damage in untreated pancreatic grafts compared with nontransplanted controls (*P* = 0.008). Among the four different treatment groups, BH4 happened to be the only compound to preserve the capillary net (Fig. 1). BH4 pretreatment of the donor (group 2) resulted in significantly higher FCD levels than in untreated grafts (group 1; *P* = 0.008). Neither NH4 (group 3) nor VitC (group 4) nor 5-MTHF (group 5) pretreatment were able to ameliorate FCD in the transplanted grafts (all, *P* = NS) and were even significantly inferior compared with BH4 (*P* = 0.008, *P* = 0.016, *P* = 0.008, respectively). FCD levels of nontransplanted controls (group 6) did not differ from BH4-treated grafts (*P* = NS; Fig. 2a).

**1:**
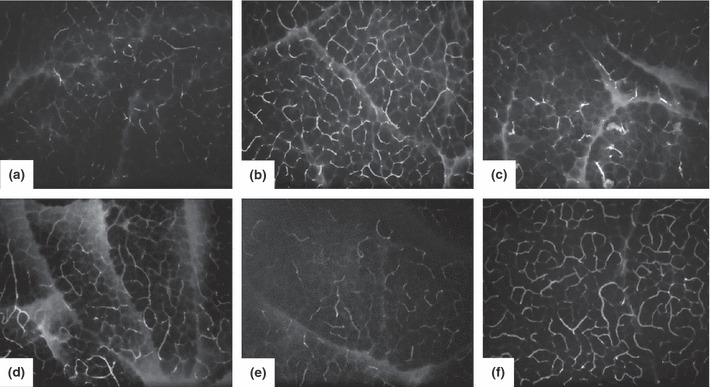
Confocal intravital fluorescence microscopy images. (a) Severe microcirculatory derangement following prolonged CIT of 16 h (untreated donors, group 1). (b) Preservation of the capillary net in BH4-pretreated animals (group 2). Failure to attenuate IRI-related microcirculatory disorder in (c) NH4-treated animals (group 3), (d) VitC-treated animals (group 4), and (e) 5-MTHF-treated animals (group 5). (f) Normal capillary perfusion of the pancreas in nontransplanted controls (group 6). Observation area: 0.00159 cm^2^ (454 μm × 349 μm).

**2:**
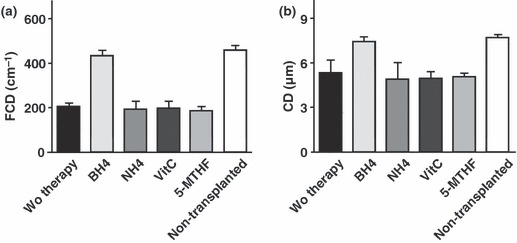
Microcirculatory parameters. (a) Tab graph representing Functional Capillary Density (FCD), defined as the length of all blood cell perfused nutritive capillaries per observation area. Data are presented as mean ± standard deviation. Statistical significances are described in detail in the main text. wo therapy: 205.0 ± 12.6 cm^−1^. BH4: 433.8 ± 22.6 cm^−1^. NH4: 192.9 ± 34.5 cm^−1^. VitC: 197.3 ± 30.5 cm^−1^. 5-MTHF: 185.4 ± 18.9 cm^−1^. Nontransplanted: 459.0 ± 19.2 cm^−1^. wo therapy = without therapy. (b) Tab graph representing Capillary Diameter (CD), defined as the mean value of the three largest capillaries per observation area. Data are presented as mean ± standard deviation. Statistical significances are described in detail in the main text, comparison between untreated animals and groups treated with NH4, VitC or 5-MTHF were not significant. wo therapy: 5.34 ± 0.86 μm. BH4: 7.44 ± 0.32 μm. NH4: 4.91 ± 1.12 μm. VitC: 4.96 ± 0.45 μm. 5-MTHF: 5.08 ± 0.24 μm. Non-transplanted: 7.71 ± 0.20 μm. wo therapy = without therapy.

Similar results were obtained for CD (Fig. 2b). While untreated grafts (group 1) resulted in significantly inferior values compared with nontransplanted controls (group 6, *P* = 0.008), CD levels following BH4 pretreatment (group 2) were similar to nontransplanted controls (group 2 vs. group 6: *P* = NS). All other treatments did not improve CD values if compared with untreated animals (all, *P* = NS).

### Graft histopathology

Most prominent necrotic formations as well as edema and inflammatory infiltrates were found in untreated transplanted grafts (group 1). Only BH4 application (group 2) resulted in a significant reduction of parenchymal edema as well as acinar and hemorrhagic fat necroses compared with group 1 (*P* = 0.007, *P* = 0.017 and *P* = 0.016, respectively). In contrast, NH4 (group 3), VitC (group 4), and 5-MTHF (group 5) substantially reduced edema formation (group 1 vs. group 3, *P* = 0.007; group 1 vs. group 4, *P* = 0.009; group 1 vs. group 5, *P* = 0.004) without, however, influencing necrotic alterations (group 1 vs. group 3, group 1 vs. group 4, group 1 vs. group 5, all *P* = NS). All treatments did not decrease inflammatory infiltrates (*P* = NS; Figs 3 and 4).

**3:**
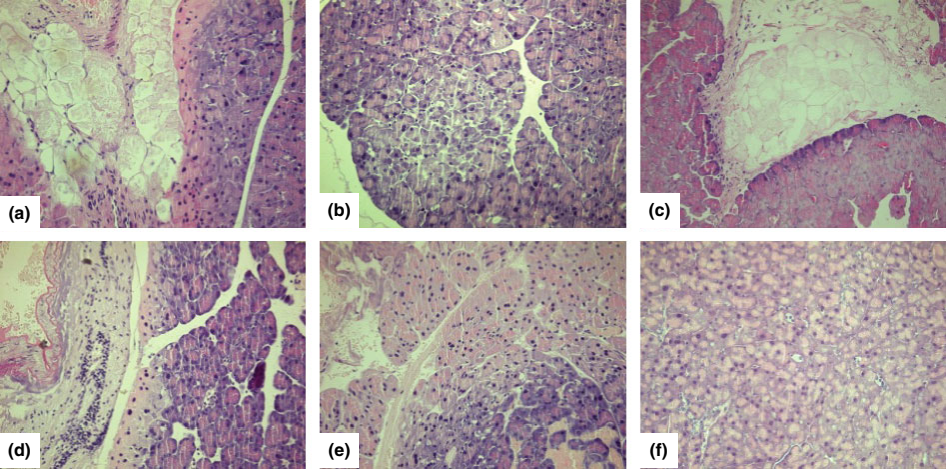
Graft histopathology (H&E staining). (a) Prominent parenchymal damage following 16-h CIT in untreated grafts (group 1). (b) Attenuation of IRI-associated parenchymal damage by BH4 pretreatment (group 2). No signs of improvement following pretreatment with NH4 (c, group 3), VitC (d, group 4), or 5-MTHF (e, group 5). (f) Nontransplanted controls (group 6) showed only slight edema.

**4:**
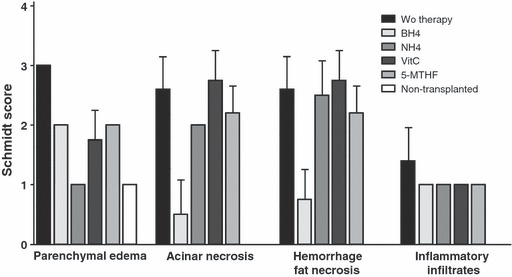
Schmidt pancreatitis score. Tab graph reporting the semiquantitative histopathological Schmidt score including parenchymal edema, acinar, hemorrhagic and fat necrosis, and inflammatory infiltrates. Data are presented as mean ± standard deviation. Statistical significances are described in detail in the main text. wo therapy = without therapy. *n* = 5 per group, three samples per graft.

### Nitrotyrosine formation

Nitrotyrosine staining indicates peroxynitrite (ONOO^−^) formation. Whereas IHC scores of untreated grafts (group 1) were strongly increased compared to nontransplanted animals (*P* = 0.004), BH4 – (group 2) and NH4 pretreatment (group 3) showed a significant reduction of nitrotyrosine formation compared with untreated grafts (group 1; *P* = 0.009 and *P* = 0.010, respectively), and were both comparable to nontransplanted controls (group 6; *P* = NS). In contrast, VitC (group 4) and 5-MTHF (group 5) did not attenuate intragraft nitrotyrosine production (*P* = 0.007 and *P* = NS, respectively, Figs 5 and 6).

**5:**
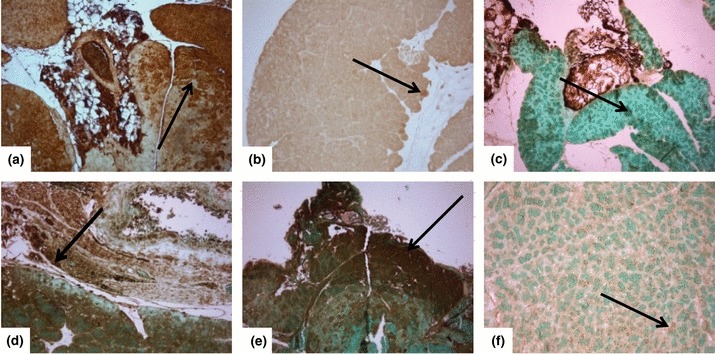
Graft nitrotyrosine immunohistochemistry. (a) Diffuse staining after 16-h CIT in untreated grafts (group 1). (b) Reduction of intensity and quantity of stained cells following BH4 pretreatment (group 2). (c) Similar improvement following NH4 pretreatment (group 3). Neither VitC (d, group 4) nor 5-MTHF (e, group 5) could reduce nitrotyrosine formation. (f) Nontransplanted controls (group 6). Arrows point to stained cells. Haemalaun blue background color was used for (a) and (b), methyl green for (c–f).

**6:**
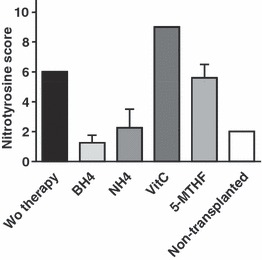
Semiquantitative immunohistochemistry score. Tab graph depicting the semiquantitative score. For quantification purposes, the product of proportion of positive cells in quartiles (0, 1, 2, 3, 4), and the staining intensity (0 no staining; 1 weak; 2 moderate; 3 strong) was calculated, yielding a total semiquantitative immunostaining score ranging from 0 to 12. Data are presented as mean ± standard deviation. Statistical significances are described in detail in the main text. wo therapy: 6.00 ± 0.00. BH4: 1.25 ± 0.50. NH4: 2.25 ± 1.26. VitC: 9.00 ± 0.00. 5-MTHF: 5.60 ± 0.89. Non-transplanted: 2.00 ± 0.00. wo therapy = without therapy. *n* = 5 per group, three samples per graft.

### Intragraft biopterin levels

Following organ procurement (time point I in Fig. 7), BH4 levels were highest if donors were treated with BH4 (group 2). Significantly lower levels were measured in untreated grafts (group 1, *P* = 0.008), as well as in grafts following pretreatment with NH4 (group 3, *P* = 0.008), with VitC (group 4, *P* = 0.008), and with 5-MTHF (group 5, *P* = 0.008). Compared with nontransplanted controls (group 6, data for nontransplanted controls not shown in Fig. 7 because only assessed at time point I) total BH4 levels in grafts pretreated with BH4 showed only a tendency to higher levels (*P* = 0.06). Similar results were observed following cold ischemia time (time point II in Fig. 7), with BH4 pretreatment (group 2) resulting in significantly higher intragraft BH4 levels compared with other groups (group 1, *P* = 0.008; group 3, *P* = 0.03; group 4, *P* = 0.008; group 5, *P* = 0.02). Before reperfusion (time point III in Fig. 7), BH4-pretreated grafts (group 2) still showed significantly higher BH4 levels if compared with untreated grafts (group 1, *P* = 0.008), however, not if compared with other pretreatment groups. Finally, 2 h following graft reperfusion (time point IV in Fig. 7), BH4 pretreatment (group 2) showed significantly higher BH4 levels compared with untreated as well as 5-MTHF pretreated grafts (group 1, *P* = 0.03; group 5, *P* = 0.02).

**7:**
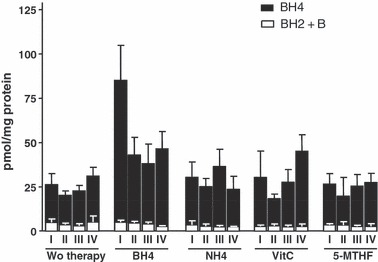
Intragraft biopterin levels. Tissues were harvested at four time points; (i), after organ procurement (*n* = 3 pro group); (ii), following cold ischemia time (*n* = 3 pro group); (iii), before reperfusion (*n* = 3 pro group); (iv), 2 h after reperfusion (*n* = 5 pro group). Black bars show BH4 levels, white bars show BH2 + B levels. Statistical significances are described in detail in the main text. wo therapy = without therapy.

BH4 represented a big percentage of the measured total intragraft biopterin, with only small amounts of dihydrobiopterin and biopterin (BH2 + B). Significant differences in BH2 + B were just observed at time point I with significantly lower levels in nontransplanted organs (group 6) compared with untreated grafts (group 1, *P* = 0.02), to BH4-pretreated grafts (group 2, *P* = 0.02), to VitC-pretreated grafts (group 4, *P* = 0.05), and to 5-MTHF-pretreated grafts (group 5, *P* = 0.02). Furthermore, pretreatment with BH4 resulted in significantly higher BH2 + B levels at time point I compared with pretreatment with VitC (*P* = 0.03).

In the NH4 treatment, neopterin derivatives remained detectable throughout all time points measured.

### Survival

Development of graft pancreatitis is known to be lethal in this model. While untreated animals survived only 2.40 ± 0.55 days, all recipients with BH4-pretreated grafts survived the whole observation period of 50 days (*P* = 0.0023). In contrast, neither pretreatment with NH4 nor with VitC nor with 5-MTHF resulted in a significant survival benefit compared with untreated animals (*P* = NS; Fig. 8).

**8:**
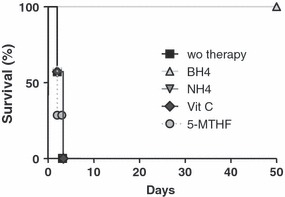
Survival analysis. Kaplan–Meier curve comparing different treatment groups with untreated animals. wo therapy versus BH4: *P* = 0.0023; wo therapy versus all other agents: *P* = NS. wo therapy = without therapy.

### Oxygen Radical Antioxidant Capacity (ORAC) assay

The ORAC assay confirmed the high antioxidative potency of BH4, which was only exceeded by NH4. In contrast, VitC and 5-MTHF showed somewhat weaker antioxidative potency (Table 2).

**2:** Oxygen radical antioxidant capacity (ORAC) assay.

Sample	Micromoles Trolox equivalent	Concentration range (μM)	Slope	Intercept	*R*^2^
BH4	1.67 ± 0.04	0.20–1.56	6.55	2.21	0.96
NH4	2.02 ± 0.03	0.20–1.56	6.61	3.44	0.94
VitC	0.87 ± 0.06	0.20–1.56	3.56	0.88	0.97
5-MTHF	1.23 ± 0.07	0.20–1.56	5.59	1.52	0.99

Comparison of ORAC values of BH4, NH4, VitC, and 5-MTHF, the concentration range measured in the ORAC assay and regression coefficients of compound concentration and ORAC activity (netAUC). Results shown are means ± standard deviation of four independent experiments done in duplicates.

### Influence of compounds on reactive oxygen species (ROS) action in cultured cells

All tested compounds showed equal capacity of scavenging ROS in cultured HepG2 cells (Fig. 9).

**9:**
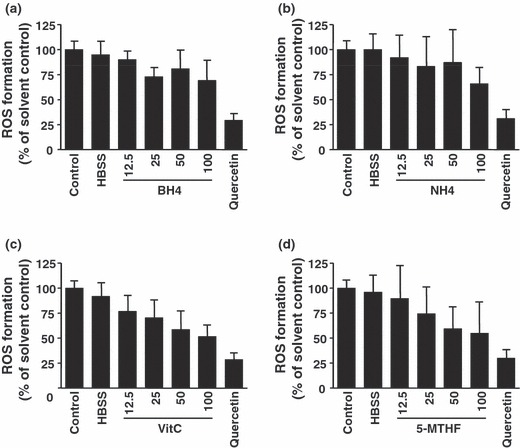
Attenuation of reactive oxygen species action in cultured cells. Inhibition of peroxyl radical (AAPH; 600 μM)-induced formation of ROS in HepG2 cells pretreated with either quercetin (10 μM), as a positive control, or increasing concentrations of BH4 (a, group 2), NH4 (b, group 3), VitC (c, group 4), and 5-MTHF (d, group 5) (12.5–100 μM). The mean percentages of DCF fluorescence, as a measure of ROS formation, are shown in relation to the AAPH-stimulated solvent control (0.5% DMSO, set to 100%). Results shown are means ± standard deviation of at least four independent experiments.

## Discussion

This study was designed to get more insight into the action of BH4 in preventing IRI. The results clearly highlight (i) that the achievement of recipient survival in this model known to be lethal is specific for BH4 and (ii) further indicate an involvement of NOS in IRI following pancreas transplantation.

We used the previously described murine pancreas transplantation model, which is known to develop severe IRI if grafts are subjected to 16-h cold ischemia time [22]. This injury results in 100% recipient lethality and directly correlates with pancreatic microcirculatory disorders and parenchymal necrosis already 2 h following graft reperfusion [12]. In contrast to the other compounds tested, only BH4 significantly improved all analyzed parameters. *In vivo* assessment of microcirculatory derangements, known to be directly related to IRI-associated graft pancreatitis [28,29], revealed that only BH4 pretreatment preserved the capillary net from disruption. In addition, only BH4-treated grafts showed significant attenuation of parenchymal damage. Inflammatory infiltrates were equal throughout the different groups, probably because of the short reperfusion period of only 2 h. Mild edema observed has to be attributed to the surgical handling during recovery. Prominent formation of the ONOO^−^ marker nitrotyrosine [30] was efficiently prevented by BH4 treatment and the significantly lower score compared with nontransplanted animals suggests an additional protection against surgical/recovery associated trauma. Even though at all four time points total biopterin tissue levels of this group were higher compared with other groups, its levels steadily decreased, reaching finally amounts comparable with other treatment agents. Hence, elevated intragraft levels already at the time of organ retrieval are crucial to prevent IRI. Confirming the results obtained 2 h following reperfusion, only recipients transplanted with BH4-treated grafts resulted in indefinite survival, the most robust readout of this model.

To draw conclusions regarding possible targets of BH4 treatment, we compared it to NH4, VitC, or 5-MTHF treatment. Using two different ROS assays, we could nicely confirm that all chosen compounds had similar or, for NH4, even higher potency of scavenging ROS compared with BH4. Failing to protect the pancreatic graft from IRI would therefore rule out the antioxidative capacity as essential element of BH4-specific protection. In addition, like BH4, also NH4 acts as cofactor of aromatic amino acid hydroxylases and alkylglycerol monooxygenase in a concentration range comparable to BH4, however, not for NOS [21]. The failure of NH4 treatment would therefore allow precluding involvement of these enzymes for the protective action and leaving only NOS as a plausible target.

Protective effects of neopterin derivates have already been described in mouse IRI models [31] as well as in a recent human study analyzing IRI in forearms of healthy volunteers [32]. However, others reported no protective effects at all of NH4 compared with BH4, using the same forearm model in chronic smokers [33]. In our model, NH4 showed equal abrogation of ONOO^−^ production like BH4, which turned out, however, to be of no benefit in terms of recipient survival. As NH4 occurs predominantly as tetrahydro derivatives in the graft, pharmacokinetic effects, explaining the different behavior of the pteridine derivatives, can be ruled out.

For VitC and 5-MTHF, the protective effect described in animal as well as in human studies [16,34,35] is thought to be mediated by increasing BH4 availability. While VitC has been described to stabilize the reduced form of BH4 [17] and to recycle the oxidized H3B^•^ radical back to BH4 [18], and decreased VitC plasma levels were also found to be associated with IRI [36], 5-MTHF has been described to facilitate donation of the electron of BH4 during the catalytic process [37] and to facilitate cofactor activity [20]. Because of the approximately 10^5^– fold less reactivity with superoxide compared with NO [38], we opted for a supraphysiological application of VitC in our model, which is, however, comparable to other published protocols [39]. Surprisingly, neither VitC nor 5-MTHF pretreatment resulted in prevention of IRI. In contrast to *in vitro* data, neither VitC nor 5-MTHF were able to increase intragraft BH4 levels, which fits however nicely with the observation of substantial NO increase and O_2_^−^ decrease following 5-MTHF application in BH4-repleted but not in BH4–free eNOS [37]. Finally, no recipient mice receiving VitC- or 5-MTHF-treated grafts survived.

Dysfunction of the constitutive NOS isoform, also referred to as “uncoupling” of the enzyme, has been directly correlated to cardiovascular pathologies like diabetes mellitus and arterial hypertension. These pathologies are associated with BH4 deficiency and physiological vascular tone could be reestablished with BH4 supplementation [33,40,41]. The common denominator with IRI is oxidative stress. A commonly used interpretation is as follows. Following the burst of oxidant agents, the fragile balance of NO production is lost. Because of its strong antioxidative capacity, BH4 captures accumulating radicals and becomes depleted. This depletion leads to uncoupling of NOS, switching its production form NO to superoxide, which in turn reacts with NO to ONOO^−^ leading finally to further oxidation and decreased bioavailability of BH4 [42]. So, BH4 treatment would mimic exogenous NO supplementation by reestablishing NO production through constitutive NOS. Although the prevention of uncoupling of NOS is our favorite hypothesis, we cannot provide an experimental proof of this hypothesis in pancreas tissue. While this model has the advantage of providing a uniquely clear readout in the form of survival, it has the disadvantage that the pancreas is full of highly active enzymes efficiently degrading protein and RNA, rendering impossible the application of techniques, such as e.g. investigation of NOS dimer formation, which allow a further characterization of BH4 effects. In particular, we were not able to detect uncoupled superoxide formation in pancreas homogenates by the dihydroethidium-HPLC assay [43], presumably because of rapid self-degradation of the tissue under assay conditions. Even though our data constrain the target of BH4 on the NOS family, further analysis of the mechanism of action was therefore not possible. However, our findings correlate with previous reports suggesting that the beneficial effect of BH4 relies on its role in enhancing the NO/cGMP pathway [44]. In most studies analyzing the role of NO in IRI following pancreas transplantation, both, NO supplementation as well as NO inhibition protected the graft [45]. Our hypothesis supports both, the observed protective effect of the NOS inhibitor L-NAME, which blocks the uncoupled enzyme [46] and also nicely fits with the graft protection achieved with NO supplementation (L-arginine or SNP administration) [47] mimicked by a functioning NOS in the presence of BH4. As in our model, graft injury occurred already within 2 h following reperfusion, the inducible isoform may, in our opinion, not be a major player on which the beneficial effect of BH4 relies on.

In conclusion, the striking superiority of tetrahydrobiopterin compared with the other compounds tested confirms the efficacy of this treatment seen in our previous studies and highlights its specificity. Further investigations will aim on the one hand at defining its mechanism of action using mouse strains with targeted deletion of each of the three NOS isoforms as well as NOS inhibitors like L-NAME and the *n*NOS-selective inhibitor S-methyl-L-thiocitrulline, and on the other hand at testing this therapeutic strategy in large-scale animals to further path the way into the clinic.

## References

[1] Sutherland D, Gruessner R, Dunn D (2001). Lessons learned from more than 1,000 pancreas transplants at a single institution. Ann Surg.

[2] Nathan DM, Fogel H, Norman D (1991). Long-term metabolic and quality of life results with pancreatic/renal transplantation in insulin-dependent diabetes mellitus. Transplantation.

[3] Gueler F, Gwinner W, Schwarz A, Haller H (2004). Long-term effects of acute ischemia and reperfusion injury. Kidney Int.

[4] Pratschke J, Weiss S, Neuhaus P, Pascher A (2008). Review of nonimmunological causes for deteriorated graft function and graft loss after transplantation. Transpl Int.

[5] Gruessner A, Sutherland D (2005). Pancreas transplant outcomes for United States (US) and non-US cases as reported to the United Network for Organ Sharing (UNOS) and the International Pancreas Transplant Registry (IPTR) as of June 2004. Clin Transplant.

[6] Sollinger H, Odorico J, Becker Y, D’Alessandro A, Pirsch J (2009). One thousand simultaneous pancreas-kidney transplants at a single center with 22-year follow-up. Ann Surg.

[7] Christoph T, Gruessner Rainer WGSDER (2004). Surgical complications. Transplantation of the Pancreas.

[8] Maglione M, Oberhuber R, Cardini B (2010). Donor pretreatment with tetrahydrobiopterin saves pancreatic isografts from ischemia reperfusion injury in a mouse model. Am J Transplant.

[9] Thoeny B, Auerbach G, Blau N (2000). Tetrahydrobiopterin biosynthesis, regeneration and functions. Biochem J.

[10] Schmidt PP, Lange R, Gorren AC, Werner ER, Mayer B, Andersson KK (2001). Formation of a protonated trihydrobiopterin radical cation in the first reaction cycle of neuronal and endothelial nitric oxide synthase detected by electron paramagnetic resonance spectroscopy. J BiolInorgChem.

[11] Watschinger K, Keller MA, Golderer G (2010). Identification of the gene encoding alkylglycerol monooxygenase defines a third class of tetrahydrobiopterin-dependent enzymes. Proc Natl Acad Sci U S A.

[12] Maglione M, Hermann M, Hengster P (2006). Tetrahydrobiopterin attenuates microvascular reperfusion injury following murine pancreas transplantation. Am J Transplant.

[13] Schulz E, Jansen T, Wenzel P, Daiber A, Münzel T (2008). Nitric oxide, tetrahydrobiopterin, oxidative stress, and endothelial dysfunction in hypertension. Antioxid Redox Signal.

[14] Yamashiro S, Kuniyoshi Y, Arakaki K, Uezu T, Miyagi K, Koja K (2006). Cardioprotective effects of tetrahydrobiopterin in cold heart preservation after cardiac arrest. Ann Thorac Cardiovasc Surg.

[15] Sucher R, Gehwolf P, Oberhuber R (2010). Tetrahydrobiopterin protects the kidney from ischemia-reperfusion injury. Kidney Int.

[16] Rodrigo R, Guichard C, Charles R (2007). Clinical pharmacology and therapeutic use of antioxidant vitamins. Fundam Clin Pharmacol.

[17] Heller R, Unbehaun A, Schellenberg B, Mayer B, Werner-Felmayer G, Werner ER (2001). L-ascorbic acid potentiates endothelial nitric oxide synthesis via a chemical stabilization of tetrahydrobiopterin. J BiolChem.

[18] Kuzkaya N, Weissmann N, Harrison D, Dikalov S (2003). Interactions of peroxynitrite, tetrahydrobiopterin, ascorbic acid, and thiols: implications for uncoupling endothelial nitric-oxide synthase. J Biol Chem.

[19] Moens AL, Vrints CJ, Claeys MJ, Timmermans JP, Champion HC, Kass DA (2008). Mechanisms and potential therapeutic targets for folic acid in cardiovascular disease. Am J Physiol Heart Circ Physiol.

[20] Hyndman ME, Verma S, Rosenfeld RJ, Anderson TJ, Parsons HG (2002). Interaction of 5-methyltetrahydrofolate and tetrahydrobiopterin on endothelial function. Am J Physiol Heart Circ Physiol.

[21] Gorren AC, Kungl AJ, Schmidt K, Werner ER, Mayer B (2001). Electrochemistry of pterin cofactors and inhibitors of nitric oxide synthase. NitricOxide.

[22] Maglione M, Hermann M, Hengster P (2007). A novel technique for heterotopic vascularized pancreas transplantation in mice to assess ischemia reperfusion injury and graft pancreatitis. Surgery.

[23] Brandacher G, Maglione M, Schneeberger S (2006). Tetrahydrobiopterin compounds prolong allograft survival independently of their effect on nitric oxide synthase activity. Transplantation.

[24] Brandacher G, Zou Y, Obrist P (2001). The 4-amino analogue of tetrahydrobiopterin efficiently prolongs murine cardiac-allograft survival. J Heart Lung Transplant.

[25] Schmidt J, Rattner D, Lewandrowski K (1992). A better model of acute pancreatitis for evaluating therapy. Ann Surg.

[26] Ou B, Hampsch-Woodill M, Prior RL (2001). Development and validation of an improved oxygen radical absorbance capacity assay using fluorescein as the fluorescent probe. J Agric Food Chem.

[27] Bass DA, Parce JW, Dechatelet LR, Szejda P, Seeds MC, Thomas M (1983). Flow cytometric studies of oxidative product formation by neutrophils: a graded response to membrane stimulation. J Immunol.

[28] Drognitz O, Obermaier R, Liu X (2004). Effects of organ preservation, ischemia time and caspase inhibition on apoptosis and microcirculation in rat pancreas transplantation. Am J Transplant.

[29] Schaser K, Puhl G, Vollmar B (2005). In vivo imaging of human pancreatic microcirculation and pancreatic tissue injury in clinical pancreas transplantation. Am J Transplant.

[30] Haddad I, Pataki G, Hu P, Galliani C, Beckman J, Matalon S (1994). Quantitation of nitrotyrosine levels in lung sections of patients and animals with acute lung injury. J Clin Invest.

[31] Icho T, Kojima S, Hayashi M, Kajiwara Y, Kitabatake K, Kubota K (1995). Suppression of ischemia-reperfusion injury in murine models by neopterins. Toxicol Appl Pharmacol.

[32] Mayahi L, Heales S, Owen D (2007). (6R)-5,6,7,8-tetrahydro-L-biopterin and its stereoisomer prevent ischemia reperfusion injury in human forearm. Arteriosclerosis Thrombosis Vascular Biol.

[33] Heitzer T, Brockhoff C, Mayer B (2000). Tetrahydrobiopterin improves endothelium-dependent vasodilation in chronic smokers : evidence for a dysfunctional nitric oxide synthase. Circ Res.

[34] Guaiquil VH, Golde DW, Beckles DL, Mascareno EJ, Siddiqui MA (2004). Vitamin C inhibits hypoxia-induced damage and apoptotic signaling pathways in cardiomyocytes and ischemic hearts. Free Radic Biol Med.

[35] Moens AL, Champion HC, Claeys MJ (2008). High-dose folic acid pretreatment blunts cardiac dysfunction during ischemia coupled to maintenance of high-energy phosphates and reduces postreperfusion injury. Circulation.

[36] Risby TH, Maley W, Scott RP (1994). Evidence for free radical-mediated lipid peroxidation at reperfusion of human orthotopic liver transplants. Surgery.

[37] Stroes ES, van Faassen EE, Yo M (2000). Folic acid reverts dysfunction of endothelial nitric oxide synthase. Circ Res.

[38] Blanchard J, Tozer TN, Rowland M (1997). Pharmacokinetic perspectives on megadoses of ascorbic acid. Am J Clin Nutr.

[39] Korkmaz A, Kolankaya D (2009). The protective effects of ascorbic acid against renal ischemia-reperfusion injury in male rats. Ren Fail.

[40] Akamine E, Kawamoto E, Scavone C (2006). Correction of endothelial dysfunction in diabetic female rats by tetrahydrobiopterin and chronic insulin. J Vasc Res.

[41] Porkert M, Sher S, Reddy U (2008). Tetrahydrobiopterin: a novel antihypertensive therapy. J Hum Hypertens.

[42] Katusic Z, d’Uscio L, Nath K (2009). Vascular protection by tetrahydrobiopterin: progress and therapeutic prospects. Trends Pharmacol Sci.

[43] Zielonka J, Vasquez-Vivar J, Kalyanaraman B (2008). Detection of 2-hydroxyethidium in cellular systems: a unique marker product of superoxide and hydroethidine. Nat Protoc.

[44] Schmid RA, Hillinger S, Walter R (1999). The nitric oxide synthase cofactor tetrahydrobiopterin reduces allograft ischemia-reperfusion injury after lung transplantation. J ThoracCardiovascSurg.

[45] Hegyi P, Rakonczay Z (2011). The role of nitric oxide in the physiology and pathophysiology of the exocrine pancreas. Antioxid Redox Signal.

[46] Hotter G, Closa D, Pi F (1995). Nitric oxide and arachidonate metabolism in ischemia-reperfusion associated with pancreas transplantation. Transplantation.

[47] Benz S, Obermaier R, Wiessner R (2002). Effect of nitric oxide in ischemia/reperfusion of the pancreas. J Surg Res.

